# Secondary Metabolites from the Culture of the Marine-derived Fungus *Paradendryphiella salina* PC 362H and Evaluation of the Anticancer Activity of Its Metabolite Hyalodendrin

**DOI:** 10.3390/md18040191

**Published:** 2020-04-03

**Authors:** Ambre Dezaire, Christophe H. Marchand, Marine Vallet, Nathalie Ferrand, Soraya Chaouch, Elisabeth Mouray, Annette K. Larsen, Michèle Sabbah, Stéphane D. Lemaire, Soizic Prado, Alexandre E. Escargueil

**Affiliations:** 1 Sorbonne Université, INSERM U938, Centre de Recherche Saint-Antoine, F-75012 Paris, France; ambre.dezaire@hotmail.fr (A.D.); nathalie.ferrand@inserm.fr (N.F.); annette.larsen@upmc.fr (A.K.L.); michele.sabbah@inserm.fr (M.S.); 2 Unité Molécules de Communication et Adaptation des Micro-organismes (UMR 7245), Muséum national d’Histoire naturelle, CNRS, F-75005 Paris, France; mvallet@ice.mpg.de (M.V.); schaouch@mnhn.fr (S.C.); mouray@mnhn.fr (E.M.); 3 CNRS, Sorbonne Université, Institut de Biologie Physico-Chimique, UMR8226, F-75005 Paris, France; christophe.marchand@ibpc.fr (C.H.M.); stephane.lemaire@ibpc.fr (S.D.L.); 4 CNRS, Institut de Biologie Physico-Chimique, Plateforme de Protéomique, FR550, F-75005 Paris, France; 5 Sorbonne Université, INSERM, Institut de Myologie, U974, Center for Research in Myology, 47 Boulevard de l’hôpital, F-75013 Paris, France

**Keywords:** anticancer agent, marine-derived fungi, secondary metabolites, resistant phenotypes, epidithiodioxopiperazines

## Abstract

High-throughput screening assays have been designed to identify compounds capable of inhibiting phenotypes involved in cancer aggressiveness. However, most studies used commercially available chemical libraries. This prompted us to explore natural products isolated from marine-derived fungi as a new source of molecules. In this study, we established a chemical library from 99 strains corresponding to 45 molecular operational taxonomic units and evaluated their anticancer activity against the MCF7 epithelial cancer cell line and its invasive stem cell-like MCF7-Sh-WISP2 counterpart. We identified the marine fungal *Paradendryphiella salina* PC 362H strain, isolated from the brown alga *Pelvetia caniculata* (PC), as one of the most promising fungi which produce active compounds. Further chemical and biological characterizations of the culture of the *Paradendryphiella salina* PC 362H strain identified (-)-hyalodendrin as the active secondary metabolite responsible for the cytotoxic activity of the crude extract. The antitumor activity of (-)-hyalodendrin was not only limited to the MCF7 cell lines, but also prominent on cancer cells with invasive phenotypes including colorectal cancer cells resistant to chemotherapy. Further investigations showed that treatment of MCF7-Sh-WISP2 cells with (-)-hyalodendrin induced changes in the phosphorylation status of p53 and altered expression of HSP60, HSP70 and PRAS40 proteins. Altogether, our study reveals that this uninvestigated marine fungal crude extract possesses a strong therapeutic potential against tumor cells with aggressive phenotypes and confirms that members of the epidithiodioxopiperazines are interesting fungal toxins with anticancer activities.

## 1. Introduction

Carcinogenesis is an evolutionary disease driven by somatic cell mutations and subclonal selection [1,2,3]. During the course of the disease, the ability of the tumor cells to adapt to a wide range of environmental conditions leads to the selection of phenotypes whose aggressiveness usually increases with the stage of cancer [3]. As an example, breast cancer progresses through defined pathological and clinical stages, starting from ductal hyperproliferation with possible evolution towards in situ and invasive carcinoma and eventually metastatic disease [4,5]. Importantly, at the in situ carcinoma stage, the tumor can be surgically removed. However, at later stages, tumor cells can undergo the epithelial-to-mesenchymal transition (EMT) [6], a biological switch associated with invasive and chemoresistant phenotypes as well as immune surveillance escape [7,8]. These observations are, however, not only limited to breast cancer, but also apply to other types of tumors such as non-small cell lung and colorectal cancers where drug resistance was associated with increased stemness and acquisition of a mesenchymal phenotype [9,10]. Altogether, these data underline the need to develop new drugs that selectively act on aggressive cancer cell behaviors.

Recently, high-throughput screening assays were designed to identify compounds capable of inhibiting phenotypes involved in cancer aggressiveness, such as EMT and cell migration [11,12]. However, most of these studies used commercially available chemical libraries. This prompted us to explore natural products isolated from marine-derived fungi as a new source of compounds capable of acting on cancer cells with mesenchymal and drug-resistant phenotypes.

Marine-derived fungi, isolated from special habitats, are a promising source for new secondary metabolites [13]. Fungi are generally considered an important source of chemical diversity, but marine-derived fungi have gained considerable attention in the last years, as many of them are structurally unique and possess interesting biological and pharmacological properties [14]. To explore the therapeutic potential of marine fungal crude extracts, we undertook a framework on the cultivable diversity associated with the brown algae *Saccharina latissima*, *Pelvetia caniculata*, *Ascophylum nodosum* and *Laminaria digitata* [15,16]. Among the different habitats colonized by marine-derived fungi, algae are indeed considered a niche of fungal diversity.

In this study, 99 strains were isolated, corresponding to 45 molecular operational taxonomic units and were cultivated on two different culture media to generate a library of crude extracts [15]. This chemical library was then evaluated against the MCF7 epithelial cancer cell line and its invasive stem cell-like MCF7-Sh-WISP2 counterpart [17,18]. This work led to the identification of the marine fungal *Paradendryphiella salina* PC 362H strain, isolated from the brown alga *Pelvetia caniculata* (PC), as one of the most promising fungi with regards to the production of active compounds. *Paradendryphiella*-related strains were previously described in the literature as producers of interesting eremophilanes and trinor-eremophilanes (dendryphiellins A–G, A1, E1, and E2), carboxylic acids (dendryphiellic acids A and B) and glyceryl ester (glyceryl dendryphiellate A) [19,20,21], as well as anthraquinone derivatives (dendryols A–D) [22]. Tricycloalternarenes were also isolated from *Paradendryphiella salina* [23]. In an effort to broaden our research for new bioactive compounds, we investigated the chemistry of the strain *Paradendryphiella salina* PC 362H.

We here report the isolation and structural characterization of the previously unreported pentanorlanostane, dendryphiellide A (**1**), together with the previously described glycocholic acid methyl ester (**2**) [24], (*3R, 6R*) hyalodendrin (**3**), an epidithiodioxopiperazine previously isolated from *Penicillium* species [25] and (*3R, 6R*) bisdethiodi(methylthio)hyalodendrin (**4**) [26]. Further biological characterization allowed the identification of **3** as the active compound responsible for the cytotoxic activity of the crude extract on MCF7 and MCF7-Sh-WISP2 cells. The antitumor activity was not limited to the MCF7 cell lines but was also prominent in cancer cells with invasive phenotypes, including colorectal cancer cells resistant to DNA targeting agents. Further investigations showed that treatment of MCF7-Sh-WISP2 cells with **3** induced changes in the phosphorylation status of p53 and altered the expression of HSP60, HSP70 and PRAS40 proteins.

Altogether, the data obtained revealed that this uninvestigated marine fungal crude extract possesses a strong therapeutic potential against tumor cells with aggressive phenotypes.

## 2. Results and Discussion

### 2.1. Cytotoxic Activity of the Crude Extracts on MCF7, MCF7-Sh-WISP2 and 3T3-F442A Cell Lines

**Figure 1:**
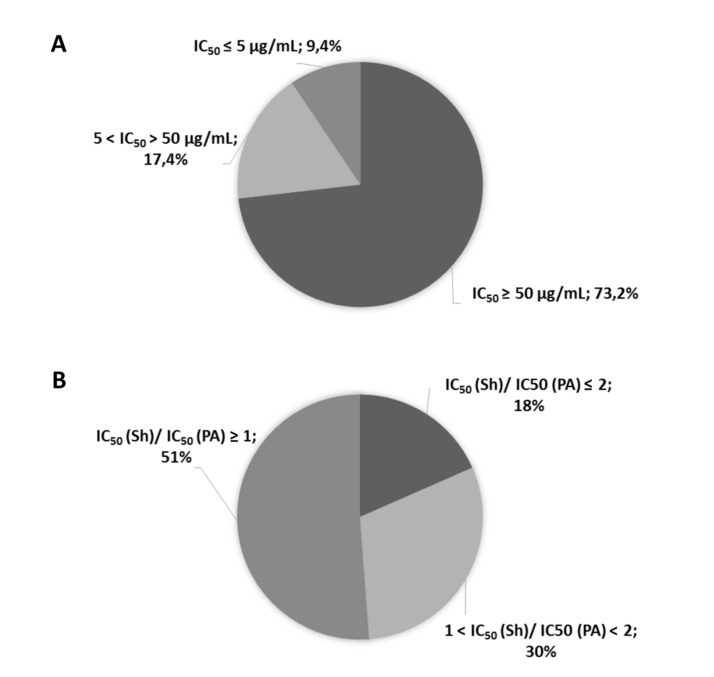
Evaluation of 138 crude extracts on MCF7-Sh-WISP2 cell viability. (**A**) Crude extracts were tested on MCF7-Sh-WISP2 cells and categorized as follows: non-active (IC_50_ ≥ 50 µg/mL); moderately active (5 < IC_50_ < 50 µg/mL) and very active (IC_50_ ≤ 5 µg/mL). IC_50_ values were calculated as the mean of two individual experiments done in duplicate. (**B**) The activity ratios of crude extracts on MCF7-Sh-WISP2 (Sh) over 3T3-F442A pre-adipocytes (PA) cell viability were calculated and were categorized as follows: mostly active on pre-adipocytes ([IC_50_ (Sh)/IC_50_ (PA)] ≥ 1); equally active on MCF7-Sh-WISP2 cells and pre-adipocytes (1 < [IC_50_ (Sh)/IC_50_ (PA)] < 2); mostly active on MCF7-Sh-WISP2 cells ([IC_50_ (Sh)/IC_50_ (PA)] ≤ 2). Among the 138 crude extracts tested, the IC_50_ (Sh)/IC_50_ (PA) ratios could not be determined for 13 of them because of out-of-range IC_50_ values for either pre-adipocytes or MCF7-Sh-WISP2 cells.

From the 99 isolates tested, 69 were able to grow enough to yield sufficient amounts of crude extracts. A cell viability assay was used to evaluate the putative anticancer activity of 138 crude extracts toward MCF7 and MCF7-Sh-WISP2 breast carcinoma cells. Murine 3T3-F442A pre-adipocyte cells were used as a control, representing non-tumorigenic cells. Among the 138 crude extracts, 13 (prepared from nine different strains) showed high cytotoxic activity against the MCF7-Sh-WISP2 cells that display an invasive phenotype (IC_50_ < 5 µg/mL) (Figure 1A and Table S1). Among the nine strains whose extracts showed high activity against MCF7-Sh-WISP2 cells, five of them showed an activity profile toward the crude extracts that was dependent on the growth media used (Table 1 and Table S1). Importantly, 25 crude extracts (18%) showed a better activity (> two-fold) towards MCF7-Sh-WISP2 compared to the 3T3-F442A pre-adipocytes (Figure 1B and Table S1). The marine fungal *P. salina* PC 362H strain produced one of the most active crude extracts when cultured on the Malt Extract Agar–Artificial Sea Water (MEA–ASW) medium with IC_50_ values of 0.4 µg/mL, 0.2 µg/mL and 0.5 µg/mL for MCF7, MCF7-Sh-WISP2 and 3T3-F442A cells, respectively (Table 1 and Table S1). This strain was selected for further chemical and biological investigation.

### 2.2. Chemical Investigation of Paradendryphiella Salina PC 362H

**Figure 2:**
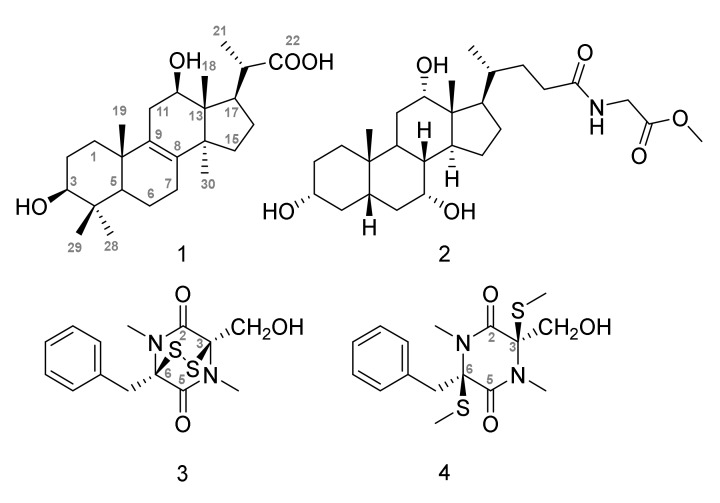
The structures of **1–4**.

**Figure 3:**
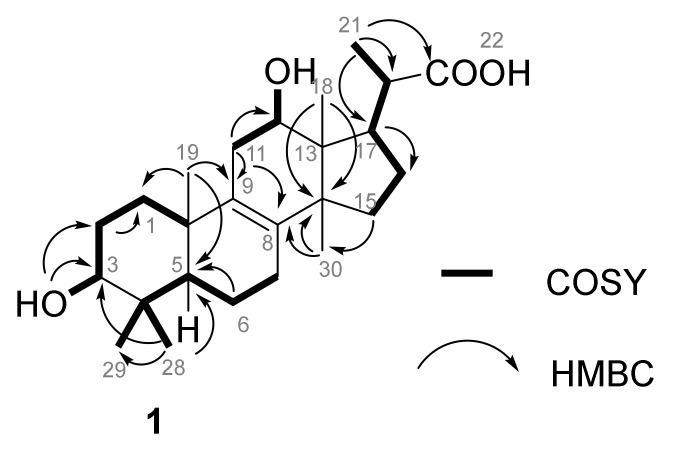
Key HMBC and COSY correlations in **1**.

Compound **1** (Figure 2) was isolated as a white powder with an [α]^20^_D_ + 5 (CH_3_CN, *c* 0.1). Its molecular formula C_25_H_40_O_4_ was established based on its (-)-HRESIMS *m/z* 403.2852 [M-H]^-^ (calculated 403.2848 for C_25_H_39_O_4_) and indicating 6 degrees of unsaturation. The IR spectrum revealed the presence of hydroxyl (3462 cm^−1^) and carbonyl (1701 cm^−1^) groups. The ^1^H NMR spectrum (Table 2) of **1** showed signals for five methyl singlets at *δ*_H_ 0.59 (3H, H-18), 0.69 (3H, H-29), 0.84 (3H, H-30), and 0.89 (3H, H-28), and 0.91 (3H, H-19), one methyl doublet at *δ*_H_ 1.21 (3H, d, *J* = 6.6 Hz, H-21), two oxymethines [*δ*_H_ 2.99 (1H, m, H-3), 3.87 (1H, t, *J* = 7.5 Hz, H-12)]. The ^13^C NMR spectrum, in combination with the HSQC spectrum, of **1** indicated the presence of 22 sp^3^ carbons: six methyl (at *δ*_C_ 9.7, 15.8, 18.9, 19.0, 23.7, 28.1), seven methylene (at *δ*_C_ 27.6, 17.8, 25.6, 25.9, 30.7, 33.6, 35.4), five methine (at *δ*_C_ 50.0, 48.1, 41.3, including two oxygen-bearing at *δ*_C_ 76.7 and 69.8) and four quaternary at *δ*_C_ 36.3, 38.6, 48.3 and 51.6 as well as two quaternary sp^2^ at *δ*_C_ 132.8 and 135.8 and one carbonyl at *δ*_C_ 178.9 (Table 2 and Figures S1 and S2). Analysis of the 2D NMR data (COSY, HSQC and HMBC, Figure 3) revealed that **1** is a pentanorlanostane. The full structural characterization revealed that **1** is closely related to the known pentanorlanostane, previously described by Xue et al. [27]. Indeed, the only difference between **1** and the previously described pentanorlanostane is the presence of a carboxylic acid in **1** instead of an ester group on C-22.

The relative configuration of **1** was deduced from NOESY correlations and the values of coupling constants. Indeed, the axial–axial coupling constant *J*_a/a_ 10.7 Hz observed in the ^1^H spectrum (DMSO) between H-3 (*δ*_H_ 2.99) and H-2ax (*δ*_H_ 1.47) established that H-3 was in an axial α-position (Table 1). This was confirmed by the NOESY correlations from OH-3 (*δ*_H_ 4.31)/H_3_-29 (*δ*_H_ 0.69)/H_3_-28 (*δ*_H_ 0.89), suggesting that OH-3 was in the equatorial β-position. In addition, the NOESY correlation between H-3 α /H_3_-28 suggested the α orientation of H_3_-28.

The relative stereochemistry of **1** was also deduced from the NOESY correlations from H_3_-19 (*δ*_H_ 0.91)/H-11β (*δ*_H_ 1.68)/H-1β (*δ*_H_ 1.60), H-11β /H_3_-18 (*δ*_H_ 0.59), H_3_-18/H-15 β (*δ*_H_ 1.58), H-15β (*δ*_H_ 1.58)/H-16β (*δ*_H_ 1.80), thus suggesting that these protons are on the same side. Conversely, the NOESY correlations from CH_3_-30 (*δ*_H_ 0.84)/H-12 (*δ*_H_ 3.87)/H-15α (*δ*_H_ 1.09)/H-17 (*δ*_H_ 1.91) indicate that CH_3_-30, H-12, H-15α and H-17 are on the opposite face.

In addition to **1**, the previously described glycocholic acid methyl ester (**2**, Figure 2) [24], (-)-(*3R, 6R*) hyalodendrin (**3**, Figure 2) [25] and (-)-(*3R, 6R*) bisdethiodi(methylthio)hyalodendrin (**4**, Figure 2), also known as gliovictin [26], were isolated from the crude extract of *P. salina* PC 362H strain. The absolute configuration of **3** was deduced from its optical rotation sign and from structural consideration. The disulfide bridge in the hyalodendrin structure indeed allows the existence of only two diasteroisomers (*3R, 6R*) or (*3S, 6S*). This, combined with the optical rotation of **3**, displaying an opposite sign compared to the natural (+)-(*3S, 6S*) hyalodendrin (Lit. [α]^D^_20_ = + 26, CHCl_3_, c 0.80) [28] and to the asymmetrically synthesized (+)-hyalodendrin [[α] ^D^_20_ = + 37] [29], suggests a (*3R, 6R*) stereochemistry for **3**. Similarly, the absolute configuration of **4** was deduced from its optical rotation sign and from the structure of **3**. In the cell, epidithiodioxopiperazines can indeed be reduced in the presence of cellular reductants and the corresponding dithiol compounds undergo bismethylation, as this was already described for bisdethiodi(methylthio)acetylaranotin [30]. Moreover, to date and to the best of our knowledge, no natural (*3R, 6S*) or (*3S, 6R*) diasteroisomers of the bisdethiodi(methylthio)hyalodendrin have been described. Together, this prompts us to propose the (*3R, 6R*) configuration for **4**.

### 2.3. Cytotoxic Activity of **1**–**4** toward MCF7, MCF7-Sh-WISP2 and 3T3-F442A Cells

The cytotoxic activity of **1** and **2** was evaluated toward MCF7, MCF7-Sh-WISP2 and 3T3-F442A cells and both compounds showed no activity at a concentration as high as 50 µM (Table 3). In contrast, **1** and **2** displayed a weak activity against *Plasmodium falciparum* with IC_50_ values of 65 ± 4.2 and 20.5 ± 3.5 µM, respectively (data not shown). Compound **4** showed only a modest activity against MCF7, MCF7-Sh-WISP2 and 3T3-F442A cells, with IC_50_ values of 42, 68 and 26 µM, respectively (Table 3). Strikingly, **3**, which possesses a disulfide bond between C-3 and C-6 instead of two methylsulfide groups (Figure 2), shows a strong activity against the MCF7-Sh-WISP2 invasive cells with an IC_50_ value of 140 nM. Interestingly, **3** is more active against MCF7-Sh-WISP2 than MCF7 or 3T3-F442A. This profile is compatible with the activity observed for the crude extract prepared from the *P. salina* PC 362H strain (Table 1). These data strongly suggest that **3** is responsible for the cytotoxic activity of the crude extract on MCF7 and MCF7-Sh-WISP2. To the best of our knowledge, this is the first report of the anticancer activity of this compound otherwise known for its anti-bacterial and anti-fungal activities [25,30,31].

### 2.4. Cell Signaling Pathways Modulated Following Exposure to **3**

**Figure 4:**
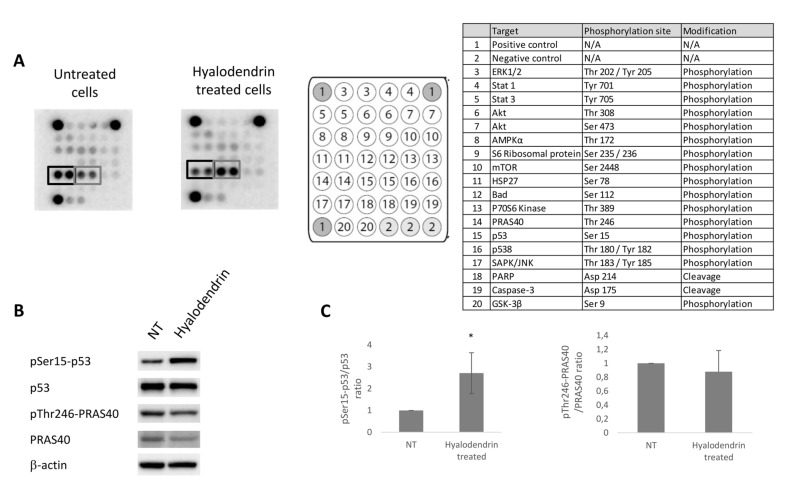
Intracellular signaling pathways modulated in MCF7-Sh-WISP2 cells exposed to **3**. (**A**) MCF7-Sh-WISP2 cells were treated or not with 2.8 µM of **3** for 48 h. The cells were lysed and the protein extracts were subjected to a protein array analysis. The table chart on the right of the blot indicates the individual proteins tested here. Results are representative of two individual experiments. (**B**) MCF7-Sh-WISP2 cells were treated or not with 2.4 µM of **3** for 72 h. The cells were then lysed and the antigens revealed by immunolabelling using antibodies directed against p53, phospho-p53 (Serine 15), PRAS40 and phospho-PRAS40 (Threonine 246). β-Actin immunoblot served as a loading control. (**C**) Western blots from three independent experiments were quantified by densitometry and values expressed as (phospho-p53/total p53) or (phospho-PRAS40/total PRAS40) ratios. The statistical analysis of experimental data was performed using Student’s paired t-test comparing the hyalodendrin-treated samples with the vehicle control. Results are expressed as means ± standard deviation (SD), * *p* < 0.05.

In order to identify pathways that are involved in the response to **3**, MCF7-Sh-WISP2 cells were treated with or without **3** (Figure 4). Cell lysates were prepared and incubated on a slide-based antibody array allowing simultaneous detection of 18 important and well-characterized signaling molecules when phosphorylated or cleaved (Figure 4A). Treatment of MCF7-Sh-WISP2 cells with **3** for 48 h led to an increased phosphorylation of p53 on serine 15. This residue is the primary target on the p53 protein of the DNA damage response (DDR) and is phosphorylated by both the ATM (Ataxia telangiectasia mutated) and ATR (ATM and RAD3-related) protein kinases [32]. Western blotting experiments confirmed the increased phosphorylation of p53 on serine 15 observed after exposure to **3** (Figure 4B,C). Treatment of MCF7-Sh-WISP2 cells with **3** also led to an apparent decrease in PRAS40 (Proline Rich Akt Substrate 40 kDa) phosphorylation on threonine 246 (Figure 4A). However, Western blotting analysis revealed that this observation was the result of a general decrease in PRAS40 protein expression levels (Figure 4B,C). Remarkably, the decrease in PRAS40 protein expression was associated with a change in PRAS40 cellular localization (Figure S3). This observation suggests that the PI3K/Akt and mTOR pathways might be involved in the anticancer activity of **3**. The cytoplasmic form of PRAS40 is indeed known to play a role in the regulation of these two signaling pathways by directly regulating the mTORC1 kinase activity [33,34].

To determine whether p53 phosphorylation on serine 15 was a result of DNA damage induced by **3**, MCF7-Sh-WISP2 cells were immuno-labelled with the γ-H2AX antibody following exposure to **3** (Figure S4). The phosphorylation of the histone variant H2AX is considered a hallmark of the DDR [35]. Interestingly, no γ-H2AX signal could be detected in cells treated for 3 h in the presence of **3**. However, after longer exposure times (24 and 48 h), cells showed a punctuated labeling in their nucleus identifying phosphorylated histone H2AX foci. Consistently, no phosphorylation of p53 on serine 15 was detected at earlier time points (data not shown). These results suggest that DNA might not be the primary target of **3** and that the lesions induced as well as the subsequent phosphorylation of p53 on serine 15 might be the result of indirect processes induced by **3**. Of note, no cleavage of either PARP or caspase-3 was observed in MCF7-Sh-WISP2 cells treated with **3** probably because MCF7 cells are defective in caspase-3 expression [36].

**Figure 5:**
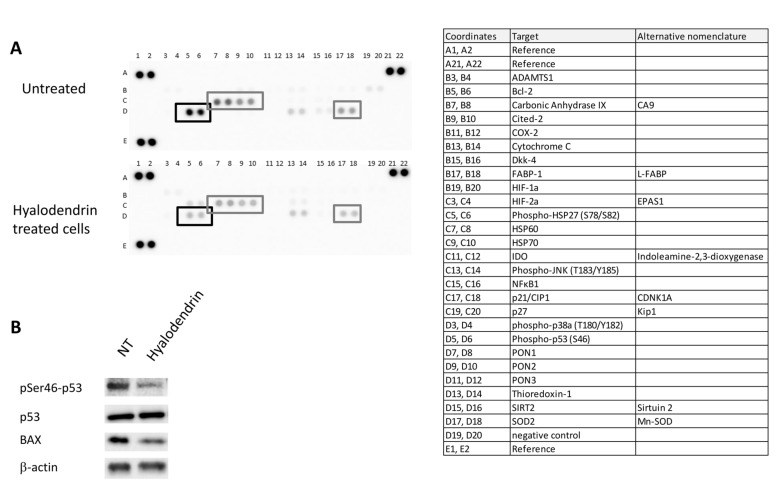
Expression of cell stress-related proteins in MCF7-Sh-WISP2 cells exposed to **3**. (**A**) MCF7-Sh-WISP2 cells were treated or untreated with 5.7 µM of **3** for 48 h. The cells were lysed and the protein extracts were subjected to a protein array analysis. The table chart on the right of the blot indicates the individual proteins tested here. (**B**) MCF7-Sh-WISP2 cells were treated or untreated with 2.4 µM of **3** for 72 h. The cells were then lysed and the antigens revealed by immunolabelling using antibodies directed against p53, phospho-p53 (Serine 46), BAX and β-actin.

To determine whether **3** might influence the expression levels of cell stress-related proteins, MCF7-Sh-WISP2 cells were exposed to **3** for 48 h. Cell lysates were prepared and analyzed by a slide-based antibody array allowing the detection of 26 human cell stress-related proteins. Surprisingly, **3**-induced phosphorylation of p53 on serine 15 was not accompanied by an increased phosphorylation of serine 46 but, rather, by a decreased phosphorylation of this residue (Figure 5A,B). Phosphorylation of this residue is generally believed to help in promoter selection and upregulation of pro-apoptotic genes [37,38]. To confirm if some of the p53-targeted genes might be affected by the loss of serine 46 phosphorylation, Western blotting experiments were conducted. Our data revealed that exposure of the MCF7-Sh-WISP2 cells to **3** led to a decreased expression of BAX (BCL2 Associated X protein), a known apoptotic target gene of p53 (Figure 5B). Consistently, no increase in p21 protein expression levels was detected on the slide-based antibody array following exposure to **3** (Figure 5A). This absence of p21 induction was accompanied by no significant changes in the cell cycle progression of MCF7-Sh-WISP2 cells exposed to **3** (Figure S5). Importantly, the decrease in phosphorylation of serine 46 did not suppress cell death induction, revealed by the sub-G1 cell population arising in response to **3** (Figure S5). These results suggest that **3**-induced cell death might be a p53-independent process in MCF7-Sh-WISP2 cells. Finally, our analysis also demonstrated that **3** induced a marked decrease in the expression levels of both HSP60 and HSP70 proteins and, to a lesser extent, of SOD2 protein (Figure 5). All of these proteins are involved in the oxidative stress response and are known to functionally interact with p53 [39,40,41,42,43].

### 2.5. Compound **3** is Efficiently Reduced by the Thioredoxin System in Vitro

**Figure 6:**
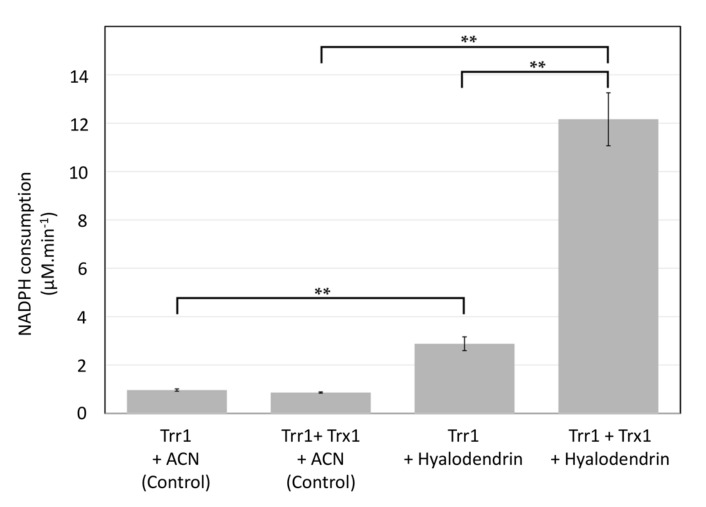
The in vitro reduction in **3** by the thioredoxin system. Compound **3,** dissolved in acetonitrile (ACN), was incubated in the presence of the NADPH thioredoxin reductase (Trr1) alone and in the presence of the whole thioredoxin system (Trr1+Trx1). NADPH consumptions were followed spectrophotometrically and compared to those obtained in control experiments (ACN alone). Results are expressed as means ± standard deviation (SD) and significance was evaluated by Student’s t-test (** *p* < 0.01).

**Figure 7:**
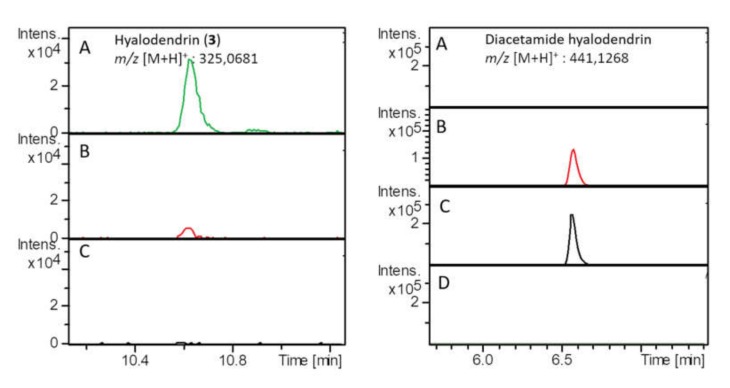
Extracted ion chromatograms of **3** (left) at *m/z* 325.0681 [M + H]^+^ and diacetamide conjugated **3** (right) at *m/z* 441.1268 [M + H]^+^ resulting from the in vitro alkylation of **3** in the presence of Trx system after addition of iodoacetamide at two different incubation times. (**A**) **3** without Trx system, (**B**) **3** in the presence of Trx system and quenched with iodoacetamide at 6 min, (**C**) **3** in the presence of Trx system and quenched with iodoacetamide at 25 min, (**D**) Trx system alone.

Compound **3** is a member of the epidithiodioxopiperazines, a class of natural products characterized by a sulfur-bridged dioxopiperazine that is believed to play an important role in their biological activity [31]. This assumption is supported by our data demonstrating that **3**, but not **4**, shows a strong anticancer activity towards the MCF7-Sh-WISP2 cells (Table 3). The role of the sulfur-bridged dioxopiperazine in the activity of epidithiodioxopiperazines has been attributed to two main mechanisms: (1) mixed disulfide formation with proteins and (2) production of reactive oxygen species (ROS) upon the formation of the disulfide bond after reduction by a cellular reductant [31,44]. Consistently, gliotoxin, an epidithiodioxopiperazine isolated in 1936 from the wood fungus *Gliocladium fimbriatum* [45] and extensively studied since then, has been shown to be capable of both inducing single and double-stranded DNA damage in the presence of cellular reductants and covalently interacting with proteins through mixed disulfide formation [46,47,48]. To test whether **3** might act through these two processes, we first assessed its propensity to form in vitro covalent adducts with the glycolytic glyceraldehyde-3-phosphate dehydrogenase enzyme (GAPDH). GAPDH is a widely used model protein to study molecular mechanism of thiol-based post-translational modifications as its catalytic cysteine is prone to multiple reversible and irreversible oxidation states [49,50]. In this context, we previously showed that cytoplasmic GAPDH from the plant model *Arabidopsis thaliana* (AtGAPDH) was able to form a mixed disulfide with the cysteine-containing tripeptide glutathione [51,52]. Using similar conditions, we here failed to observe, by Matrix-Assisted Laser Desorption–Ionization Time-of-Flight (MALDI-TOF) mass spectrometry, any mass shift of the AtGAPDH protein after incubation with **3**, indicating that **3** is not able to form a mixed disulfide with the highly reactive catalytic cysteine of AtGAPDH (data not shown). This result suggests that **3** is not primarily acting through the disulphide bridge. However, this negative result does not completely rule out the possibility of mixed-disulfide formation with specific protein substrates since the reactivity of a given cysteine with small molecules is largely dependent on its three-dimensional microenvironment in the folded protein [53]. In order to explore the second mechanism of epidithiodioxopiperazines’ toxicity, we evaluated the capacity of **3** to be enzymatically reduced by the ubiquitous thioredoxin reduction system which is composed of the thioredoxin (Trx) itself and the NADPH-dependent thioredoxin reductase (NTR). Trxs are small ubiquitous thiol/disulfide oxidoreductases with multiple recognized roles in cellular processes and human diseases including cancers [54,55,56]. However, since mammal NTR is a selenoprotein that is not easily produced recombinantly, we used here the thioredoxin system from the eukaryotic yeast *Saccharomyces cerevisiae* (Figure 6). Our data show that, in the presence of **3**, NADPH consumption increased by ca. three and 14 folds for the yeast NTR (Trr1) alone and the reconstituted thioredoxin system (Trr1+Trx1), respectively. Therefore, although Trr1 alone exhibits a low activity towards **3**, the reduction rate is strongly enhanced in the presence of Trx1. To confirm that NADPH consumption is directly linked to the reduction in the disulfide bond present in **3**, we quenched the reduction assays by adding either iodoacetamide (Figure 7) or *N*-ethylmaleimide (Figure S6), two different thiol alkylating agents, at two different incubation times (6 and 25 min). Alkylation of nascent thiols after Trx reduction indeed impairs formation of the disulfide bond. Moreover, these modifications can be easily followed by high-resolution accurate mass spectrometry, as they generate +114 Da (iodoacetamide) or +250 Da (*N*-ethylmaleimide) mass increases for the reduced form of **3**. Extracted ion chromatograms for **3** and its alkylated forms in the presence of the Trx system confirmed that the peak corresponding to **3** disappeared progressively and peaks corresponding to the alkylated forms increased with incubation time (Figure 7 and Figure S6). Consistently, no change was observed when the Trx system was omitted (Figure 7D and Figure S6D).

Taken together, these results clearly show that the disulfide bond present in **3** can be efficiently reduced by Trx1 in vitro. This suggests that the thioredoxin system could play a similar role in human cells and serve as a prime candidate for a cellular reductant for catalyzing the reduction step in vivo and thereby sustains, through redox cycling, catalytic ROS production in the presence of **3**. This result, combined with the observed phosphorylation of the histone variant H2AX and p53 on serine 15, supports the hypothesis that **3** exposure may promote the production of ROS leading to subsequent DNA breakages and activation of the DDR [57].

### 2.6. Compound **3** is Active Against a Panel of Cancer Cell Lines with Aggressive Phenotypes

We next investigated whether the activity of **3** was limited only to MCF7 cells. To do so, we first tested the activity of **3** on the triple negative (ER−/PR−/HER2−) breast cancer cell line MDA-MB-231. These cells are known to overexpress the epidermal growth factor receptor 1 (HER-1) [58]. The overexpression of HER-1 has been associated with poor prognosis, enhanced metastatic potential, and both chemoresistance and radioresistance [59]. Compound **3** showed a similar activity towards MDA-MB-231 as for the invasive triple negative MCF7-Sh-WISP2 (Table 4). This demonstrates that **3** is active on both luminal (MCF7 and MCF7-Sh-WISP2) and basal (MDA-MB-231) breast carcinoma cells, irrespective of their hormonal or HER-1 receptor status. Of note, our results also show that **3** can circumvent resistance to hormonotherapy acquired through the loss of both estrogen and progesterone receptors (Table 3, comparison of MCF7 and MCF7-Sh-WISP2 sensitivity towards **3** and hydroxytamoxifen). Therefore, our data indicate that **3** is active against breast cancer cells with invasive and drug resistant features.

To determine whether **3** is also active in cancer cells other than breast carcinoma, its activity was tested on the HeLa cervix carcinoma cell line and a panel of colorectal cancer cells with different genetic and phenotypic profiles (Table 4). Our results demonstrated that **3** is active in cancer cells originating from different organs. Most importantly, our data show that colorectal cancer cells are sensitive to **3** independent of their MSI/MSS, BRAF WT/BRAF mutated, KRAS WT/KRAS mutated and TP53 WT/TP53 mutated status. The last observation is in agreement with our result demonstrating that **3**-induced cell death in MCF7-Sh-WISP2 cells is a p53-independent process (Figure 5B and Figure S5). Moreover, cells that developed resistance to the DNA targeting agents associated with an increased stemness/mesenchymal phenotype (5-FU, oxaliplatin and SN-38) [10] still remained sensitive to **3**. Strikingly, all cancer cells showed an IC_50_ value below the values determined for both the murine 3T3-F442A pre-adipocyte cells and the human C19 immortalized fibroblasts, suggesting that **3** might preferentially target tumor cells (Table 4). Finally, the broad array of IC_50_ values calculated, ranging from 40 nM (DLD1) to 305 nM (3T3-F442A), suggests that specific biomarkers should be identified and thus help to predict the cellular response to **3**.

Therefore, the activities of **3** toward cancer cells with aggressive phenotypes (e.g., drug resistance, stem cell-like) warrant additional studies, in particular on the contributive role of the human thioredoxin system, considering the need for new bioactive compounds that act against these diseases.

## 3. Materials and Methods

### 3.1. General Experimental Procedures

Optical rotations were measured using a Perkin Elmer 341 Polarimeter. IR spectra were taken on a Shimadzu FTIR-8400S infrared spectrophotometer. Ultraviolet (UV) spectra were recorded on a Kontron Uvikon 9X3W Double Beam UV/Vis spectrophotometer (Bioserv, France). Mass spectra were recorded with a Bruker maxis II Electron Transfer Dissociation (ETD). For the collision-induced dissociation (CID) spectra, the collision energy was 40 eV and the collision gas was nitrogen. The NMR experiments were recorded on Bruker Avance III HD 300 MHz, 400 MHz, 500 MHz and 600 MHz spectrometers (Wissembourg, France) equipped with a BBFO Plus Smartprobe and a triple resonance TCI cryoprobe, respectively. Chemical shifts are expressed in δ (ppm), and are referenced to the residual non-deuterated solvent signals. Preparative High-Performance Liquid Chromatography (HPLC) was performed on an Agilent system (Technologies 1260 infinity) and an Agilent PrepHT XDB-C18 column (21.2 × 150 mm i.d.; 5 µm; USA). Column chromatography (CC) was performed using silica gel (Geduran Si 60, 40−63 µm, Merck, Germany and Lichroprep RP-18, 40−63 µm, Merck KGaA, Germany) and Sephadex LH-20 (Sigma-Aldrich Lipophilic Sephadex, Germany). Silica gel-precoated plates (F254, 20 × 20 cm, Merck KGaA, Germany) were used for thin-layer chromatography (TLC).

### 3.2. Crude Extracts Library

A fungal library was established and maintained at 4 °C on Malt Extract Agar–Artificial Sea Water (MEA–ASW) and at −80 °C in glycerol. Strains were thus inoculated in two 12 cm^2^ Petri dishes containing 60 mL of MEA–ASW or Tubaki media. The 1 L of MEA–ASW medium consists of 200 mL of distilled water, 800 mL of ASW, 20 g of malt extract, 20 g of D-glucose, 1 g of bactopeptone and 20 g of Agar. The 1 L of Tubaki-ASW medium consists of 200 mL of distilled water, 800 mL of ASW, 1 g of yeast extract, 30 g of D-Glucose, 1 g of bactopeptone, 1 g of K_2_HPO_4_, 0.5 g of MgSO_4_, 0.01 g of FeSO_4_ and 20 g of Agar. ASW was prepared as described by Kester [60]. The inoculation was made with spore suspension (100 µL, 10^4^ spores. mL^−1^) or from crushed mycelial suspension (1 cm^2^ in 2 mL of sterile ASW) for non-sporulating fungi in two 12 cm^2^ plates containing 60 mL of MEA–ASW or Tubaki. After incubation at 18 °C for 21 days, the fungal culture (mycelium and agar) were cut in 1 cm^2^ pieces and extracted with EtOAc (3 × 80 mL) for 3 × 1 h under sonication. The organic phases were combined and filtered, dried over anhydrous MgSO_4_ and concentrated under reduced pressure to yield crude extracts. A total of 138 extracts were then solubilized in DMSO at 50 mg/mL before being sampled in 96-well plates.

### 3.3. Fermentation, Extractions and Purification

*Paradendryphiella salina* PC 362H was cultured at 18 °C on MEA–ASW for 21 days using 150 Petri dishes containing 60 mL of medium. After incubation, the culture was extracted with EtOAc (3 × 5 L) and the combined organic extracts were evaporated and dried under reduced pressure, giving 11.47 g of crude extract. The EtOAc extract was subjected to flash column chromatography (CC) over silica gel (345 g) with a gradient elution using cyclohexane/EtOAc (9:1 to EtOAc neat, v/v) and EtOAc/MeOH gradient (9:1 to MeOH neat, v/v), followed by MeOH/AcOH (10:0.1, v/v) to yield 34 fractions leading to **4** (bisdethio(methylthio)hyalodendrin) (Fraction 9, 251.53 mg). Fr. 7 (295 mg) was subjected to a Sephadex LH20 column (29.5 g) and eluted with MeOH and yielded 19 fractions that were combined into four fractions (Fr.7A–Fr.7D). Compound **3** (38.9 mg) was isolated from fraction 7D (80 mg) after a flash column chromatography (CC) over silica gel (140 mg) with a gradient elution using cyclohexane/EtOAc (3:1 v/v). Compounds **1** and **2** were purified from Fr. 23 (125 mg) by semi-preparative HPLC (Agilent Technologies 1260 infinity) equipped with a diode array detector and a C18 Eclipse XDB column (21.2 × 150mm, 5μm). Mobile phases were (A): 95% milliQ water (0.05% trifluoroacetic acid), 5% acetonitrile and (B): 5% milliQ water (0.05% TFA), 95% acetonitrile. The separation was achieved at a flow rate of 10 mL/min with the following gradients: 95%–65% A; 5%–35% B in 6 min, 65%–60% A; 35%–40% B in 30 min, 60%–5% A; 40%–95% B in 3 min, 5% A; 95% B during 3 min and 5%–95% A; 95%–5% B in 6 min to yield **1** (7 mg, 23 min, 36% ACN in H_2_O) and **2** (34 min, 38% ACN in H_2_O) [24].

Dendryphiellide A (**1**): white powder; IR (NaCl) ν_max_ cm^-1^: 3462; 2941; 1672; 1558; 1209; 1022. ^1^H and ^13^C NMR data (DMSO) see Table 2; (-)-HRESIMS: *m/z*: 403.2852 [M-H]^-^ calculated for C_25_H_39_O_4_, 403.2848.

(*3R, 6R*) Hyalodendrin (**3**): [α]^20^_D_: -20 (CH_3_OH, *c* 1.0).

(*3R, 6R*) bisdethiodi(methylthio)hyalodendrin (**4**): [α]^20^_D_: -35 (CH_3_OH, *c* 1.0).

The ^1^H and ^13^C NMR spectra of **2**–**4** as well as specific rotations are compatible with those previously reported (Tables S2 and S3) [24,25,26].

### 3.4. Antibodies

Antibodies directed against PRAS40 (# 701075) and phospho-PRAS40 (Thr 246) (# 701058) were purchased from Life Technologies (Carlsbad, California). Antibodies against phospho-p53 (Ser 15) (# 9286), phospho-p53 (Ser 46) (# 2521) and BAX (# 2772) were from Cell Signaling (Ozyme, Saint Quentin en Yvelines, France). Antibodies directed against p53 (# Sc-126) and β-actin (# Sc-1616) were purchased from Santa Cruz Biotechnology. Horseradish peroxidase (HRP)-conjugated antibodies were obtained from Jackson ImmunoResearch (Bar Harbor, ME).

### 3.5. Cells

MCF7 (ER+/PR+/HER2−) and MDA-MB-231 (ER−/PR−/HER2−) human breast carcinoma cell lines as well as the 3T3-F442A pre-adipocyte cell line were obtained from American Type Culture Collections (ATCC, VA, USA). MCF7-Sh-WISP2 (ER−/PR−/HER2−) cells were kindly provided by Michèle Sabbah (Paris, France) [17,18]. HeLa-M cervical carcinoma cells were a gift from Andrzej Skladanowski (Gdansk, Poland), while colorectal carcinoma cells were kindly provided by Richard Camalier (Division of Cancer Treatment and Diagnosis Tumor Repository, National Cancer Institute) and Richard Hamelin (Paris, France). The 5-fluorouracil, SN-38 (7-Ethyl-10-hydroxycamptothecin) and oxaliplatin resistant HT29 and HCT116 cell lines were a gift from Annette K. Larsen (Paris, France) [10,61]. C19 fibroblasts were kindly provided by the platform for the immortalization of human cells from the “Institut de Myologie” (Myoline platform, Hôpital Pitié-Salpétrière, Paris, France).

Human breast carcinoma cells, immortalized fibroblasts and cervical carcinoma cells were maintained in Dulbecco’s modified Eagle’s medium (DMEM) with 10% fetal bovine serum (FBS), 100 units/mL penicillin and 100 µg/mL streptomycin (Gibco, Life Technologies). The 3T3-F442A cells were maintained in DMEM supplemented with 10% DS (Donor Serum), 200 units/mL penicillin and 200 µg/mL streptomycin, 176.4 µM biotine and 85 µM pantothenic acid. Colon carcinoma cell lines were maintained in DMEM (except for HCT116 cell lines that were cultured in McCoy medium) supplemented with 5% FBS and 100 units/mL penicillin and 100 µg /mL streptomycin.

### 3.6. Viability Assays

Cellular viability was determined by the MTT (methylthiazolyldiphenyl-tetrazolium bromide) assay. Briefly, cells were exposed to the indicated concentrations of crude extracts or compounds 1–4 for five doubling times and then incubated for 3 h at 37 °C with MTT. Formazan crystals were then dissolved in DMSO and optical density measured at 570 nm in a microplate reader. The half-maximal inhibitory concentration (IC_50_) was determined. Values given for crude extracts are averages of at least two experiments done in duplicate. IC_50_ values calculated for the four isolated compounds and the commercially available 4-hydroxy-tamoxifen (Sigma-Aldrich) are averages of at least three experiments done in duplicate.

### 3.7. Compound **3** Concentrations

To expose cells to relevant amounts of **3** in each of the experimental settings tested below, drug concentrations were adjusted for each condition (cell number, experimental volume) in order to keep the ratio of **3** per cell close to the ratio calculated from the cell viability assays. In the latter, the IC_50_ value calculated for MCF7-Sh-WISP2 cells was equal to 140 nM, corresponding to 3.8 × 10^−5^ nmol of **3** per cell.

### 3.8. Intracellular Signaling Array

MCF7-Sh-WISP2 cells were treated for 48 h with 2.8 µM of **3** (corresponding to 3.8 × 10^−5^ nmol of drug per cell), harvested and lysed on ice for 5 min with 0.5 mL of cell lysis buffer containing a cocktail of protease inhibitors. The lysates were centrifuged at 14,000 g for 1 min at 4 °C. Intracellular signaling molecules were detected using a PathScan® intracellular signaling array kit (# 7323, Cell Signaling Technology) according to the manufacturer’s procedure. The chemiluminescent images of the slide were captured with the Chemidoc system (Biorad).

### 3.9. Human Cell Stress Array

MCF7-Sh-WISP2 cells were treated for 48 h with 5.7 µM of **3** (corresponding to 3.8 × 10^−5^ nmol of drug per cell), harvested and lysed for 30 min on ice with 0.4 mL of cell lysis buffer containing a cocktail of protease inhibitors. The lysates were centrifuged at 14,000 *g* for 5 min at 4 °C. Human cell stress proteins were detected using the Proteome Profiler human cell stress array kit (# ARY018, R&D systems) according to the manufacturer’s instructions. The chemiluminescent images of the slide were captured with the Chemidoc system (Biorad).

### 3.10. Immunoblotting

MCF7-Sh-WISP2 cells were incubated for 72 h at 37 °C with 2.4 µM of **3** (corresponding to 3.8 × 10^−5^ nmol of drug per cell). Cells were then washed twice with Phosphate-Buffer Saline (PBS) 1X and lysed for 30 min in RIPA buffer (150 mM NaCl, 1.0% IGEPAL® CA-630, 0.5% sodium deoxycholate, 0.1% SDS, 50 mM Tris, pH 8.0). Proteins (20 µg) were resolved on sodium dodecyl sulfate polyacrylamide gel (SDS/PAGE) (12%) and blotted onto nitrocellulose membranes (Bio-Rad). Membranes were saturated with either PBST-milk or PBST-BSA (PBS, 0.5% Tween 20 complemented with 5% dehydrated skimmed milk or 5% Bovine Serum Albumin) for 1 h at room temperature, and the antigens were revealed by immunolabeling. Antigens were detected using an enhanced chemiluminescence kit (Biorad) using the Chemidoc system (Biorad). Protein quantitation was calculated using Image Lab software developed by Bio-Rad.

### 3.11. In Vitro Reduction in Hyalodendrin (**3**) by the Thioredoxin System

Thioredoxin 1 (Trx1) and NADPH-dependent thioredoxin reductase (Trr1) from *Saccharomyces cerevisiae* were cloned and produced as described previously [62]. Thioredoxin activity was measured spectrophotometrically (Uvikon XS; Secomam) in 1 mL cuvette by following NADPH consumption at 340 nm over 2 min. The reaction was performed in 100 µM NADPH, 246 nM of Trr1 in 30 mM Tris-HCl buffer (pH 7.9) and in the presence or not of 65 nM of Trx1. The reaction was initiated by adding 10 µL of 50 mM of **3** in acetonitrile or 10 µL of acetonitrile (ACN, Control experiment). For each condition, NADPH consumption experiments were performed in triplicate and results were represented as the mean ± SD. The significance of the results was evaluated by Student’s *t*-test.

### 3.12. High-resolution Accurate-Mass (HRMS) Mass Spectrometry

Compound **3** reduction experiments were performed as described above, except that after 6 or 25 min of incubation time, 20 µL of 200 mM iodoacetamide or 200 mM N-ethylmaleimide thiol alkylating agent were added to quench the reduction. After a 30 min extra alkylation time, samples were filtrated using centrifugal filters (Amicon-Ultra, Millipore, 3 kDa molecular weight cut-off) to remove proteins. Filtrates were frozen and kept at −20 °C until analysis by mass spectrometry. Samples were recorded on Dionex Ultimate 3000 HPLC system coupled with a Maxis II ™ Quadrupole Time-Of-Flight (QTOF) mass spectrometer (Bruker, MA, USA) fitted with an electrospray ionization (ESI) source. Separation was performed on a C18 Acclaim^TM^ Rapid Separation Liquid Chromatography (RSLC) Polar Advantage II (2.1 × 100 mm, 2.2 µm pore size) column (Thermo Scientific, MA, USA) at 40 °C. The mobile phase consisted of a mix of H_2_O + 0.1% formic acid (solvent A) and acetonitrile + 0.1% formic acid (solvent B). Injection volume was set to 2 μL and elution flow to 0.3 mL.min^−1^. The elution gradient profile was programmed as follows: 5% B for 2 min, increased up to 50% B from 2 to 9 min and to 90% B from 9 to 15 min, followed by an isocratic step of 90% B for 2 min. The initial conditions were gradually recovered from 17 to 19 min, and held for 3 min for column equilibration for a total runtime of 21 min. In the first half a minute of each run, a sodium formate solution was injected directly as an internal reference for calibration. The acquisition parameters of the ESI source were set as follows: electrospray voltage for the ESI source: 3500 V, nebulizing gas (N_2_) pressure: 35 psi, drying gas (N_2_) flow: 8 mL.min^−1^, and drying temperature: 200 °C. Mass spectra were recorded in positive ionization mode over the *m/z* range 100–1300 at a frequency of 2 Hz. For MS/MS analysis, the cycle time was 3 seconds.

## 4. Conclusions

In summary, our study reveals that hyalodendrin is an interesting fungal toxin with anticancer activity. Even if its use might be limited by its toxicity, the unique nature of the internal disulfide bond makes this compound, as well as other members of the epidithiodioxopiperazines, interesting for the design of future chemotherapeutic agents. In particular, their ability to actively kill cells with raised glutathione levels may suggest strategies to selectively address tumor cells and particularly drug-resistant cancer cells which often show high concentrations of glutathione-associated enzymes [63,64].

## Figures and Tables

**Table 1:** The most active strains for which one of the crude extracts has an IC_50_ value lower than 5 µg/mL toward MCF7-Sh-WISP2 cells. Crude extracts were assessed on cell viability using the methylthiazolyldiphenyl-tetrazolium bromide (MTT) assay. Results are expressed as means of two individual experiments.

		IC_50_ (µg/mL)				IC_50_ (µg/mL)		
Strain Code	Culture Medium	MCF7	MCF7-Sh- WISP2	3T3-F442A	Culture Medium	MCF7	MCF7-Sh- WISP2	3T3-F442A
LD150F	MEA–ASW	0.3	2.7	6.3	Tubaki	2.4	3.4	36.8
AN325T		1.5	0.4	3.7		1.8	1.2	3.8
LD327H		0.9	0.3	0.7		0.5	0.1	0.1
SL332T		4.2	3.0	3.3		61.0	111.2	50.7
PC362H		0.4	0.2	0.5		8.3	10.0	16.7
SL469T		88.2	22.8	37.6		2.3	2.8	4.7
LD481H		0.3	0.4	0.3		0.3	0.9	0.2

**Table 2:** ^1^H and ^13^C NMR data of **1** (DMSO-*d6*, 600 MHz and 150 MHz, 298K).

Pos.	δ_C_, Type	δ_H_ *Mult.* (*J* in Hz).	Pos.	δ_C_, Type	δ_H_ *Mult.* (*J* in Hz)
**1**	35.4, CH_2_	(α) 1.14, m; (β) 1.60, m	**15**	30.7, CH_2_	(α) 1.09, m; (β) 1.58, m
**2**	27.6, CH_2_	1.47, m	**16**	25.9, CH_2_	(α) 1.54, m, (β) 1.80, m
**3**	76.7, CH	2.99, dt (10.7, 5.1)	**17**	48.1, CH	1.91, m
**4**	38.6, C	-	**CH_3_-18**	9.7, CH_3_	0.59, s
**5**	50.0, CH	0.93,d (1.9)	**CH_3_-19**	18.9, CH_3_	0.91, s
**6**	17.8, CH_2_	(β) * 1.40, m; (α) * 1.63, m	**20**	41.3, CH	2.36, m
**7**	25.6, CH_2_	1.95, m	**CH_3_-21**	19.0, CH_3_	1.21, d (6.6)
**8**	132.8, C	-	**22**	178.9, C	-
**9**	135.8, C	-	**CH_3_-28**	28.1, CH_3_	0.89, s
**10**	36.3, C	-	**CH_3_-29**	15.8, CH_3_	0.69, s
**11**	33.6, CH_2_	(α) 2.38, m; (β) 1.68, m	**CH_3_-30**	23.7, CH_3_	0.84, s
**12**	69.8, CH	3.87, brt (7.5)	**OH-3**	-	4.31, d (5.1)
**13**	48.3, C	-	**OH-12**	-	4.42, br s
**14**	51.6, C	-			

Broad singlet (br s); interchangeable (*); broad signal (br).

**Table 3:** Evaluation of **1–4** on cell viability of MCF7, MCF7-Sh-WISP2 and 3T3-F442A cells. Results are shown as three individual experiments done in duplicate ± SD.

Cell Line	IC_50_ µg/mL (µM)				
	1	2	3	4	Hydroxi Tamoxifen
MCF7	20 µg/mL (50 µM)	> 25 µg/mL (> 50 µM)	0.07 µg/mL (0.22 µM)	15 µg/mL (42 µM)	8.8 µM
MCF7-Sh-WISP2	> 25 µg/mL (> 60 µM)	> 25 µg/mL (> 50 µM)	0.046 µg/mL (0.14 µM)	24 µg/mL (68 µM)	11.6 µM
3T3-F442A	> 25 µg/mL (> 60 µM)	> 25 µg/mL (> 50 µM)	0.099 µg/mL (0.3 µM)	92 µg/mL (26 µM)	14.5 µM

**Table 4:** Cytotoxic activity of **3** on a panel of cancer and non-tumorigenic cell lines. Results are shown as three individual experiments done in duplicate ± SD. Not determined (ND).

	Cell line	IC_50_ (nM)	Phenotypes	Relevant Genetic Status	MSS vs MSI
Breast	MC7-Sh-WISP2	142.0 ± 3.0	mesenchymal, invasive	ER-/PR-/HER2-	
	MCF7	216.3 ± 6.0	epithelial	ER+/PR+/HER2-, TP53 WT	
	MDA-MB-231	132.5 ± 13.4	mesenchymal, invasive	ER-/PR-/HER2-, TP53 mutant (R280K)	
Colon	SW48	149.0 ± 15.5	mesenchymal, invasive	BRAF and KRAS WT, TP53 mutant (R248Y)	MSI
	DLD1	40.0 ± 5.8	epithelial	KRAS mutant (G13D), TP53 mutant (S241F)	MSI
	HT29	58.0 ± 13.7	epithelial	BRAF mutant (V600E), TP53 mutant (R273H)	MSS
	HT29 5FU	146.8 ± 10.2	mesenchymal, invasive	ND	MSS
	HT29 oxa	141.8 ± 2.6	epithelial	ND	MSS
	HT29 SN-38	93.8 ± 13.6	epithelial	ND	MSS
	HCT116	48.0 ± 9.3	epithelial	KRAS mutant (G13D), TP53 WT	MSI
	HCT116 5FU	72.0 ± 10.9	mesenchymal, invasive	ND	MSI
	HCT116 oxa	25.7 ± 4.2	mesenchymal, invasive	ND	MSI
	HCT116 SN-38	43.8 ± 4.2	mesenchymal, invasive	ND	MSI
	LS513	78.0 ± 9.3	epithelial	KRAS mutant (G12D), TP53 WT	MSS
	LOVO	73.4 ± 16.4	epithelial	KRAS mutant (G13D; A14V), TP53 WT	MSI
	RKO	74.3 ± 1.9	mesenchymal, invasive	BRAF mutant (V600E), TP53 WT	MSI
	LS174T	158.0 ± 13.0	epithelial	KRAS mutant (G12D), TP53 WT	MSI
	SW480	163.7 ± 11.0	mesenchymal, invasive	KRAS mutant (G12V), TP53 mutant (R273H)	MSS
Cervix	HeLa	69.0 ± 9.9	epithelial	TP53 WT (HPV positive)	
Normal	3T3F442A	305.0 ± 4.2	pre-adipocytic		
	C19	252.5 ± 20.5	fibroblastic		

## Supplementary Materials

The following are available online at https://www.mdpi.com/1660-3397/18/4/191/s1, Supplementary materials and methods: Fungal material, Anti-plasmodial assay and Immunofluorescence, Table S1: Evaluation of the 138 crude extracts on MCF7-Sh-WISP2 cell viability, Table S2: ^1^H and ^13^C NMR data of **2** in CD_3_OD (MHz), Table S3: NMR spectra (^1^H and ^13^C) for **3** and **4** in CDCl3, Figure S1: ^1^H spectrum of **1** in DMSO (600 MHz), Figure S2: DEPT 135 spectrum of **1** in DMSO (150 MHz), Figure S3: Subcellular localization of PRAS40 in MCF7-Sh-WISP2 cells exposed or not to compound **3**, Figure S4: γ-H2AX foci formation in MCF7-Sh-WISP2 cells exposed or not to compound **3**, Figure S5: Cell cycle progression of MCF7-Sh-WISP2 cells in response to compound **3** exposure, Figure S6: Extracted ion chromatograms of **3** (hyalodendrin) and N-ethylmaleimide conjugated compound **3** (N-ethylmaleimide hyalodendrin), Figure S7: HSQC spectrum of **1** in DMSO (600 MHz), Figure S8: HMBC spectrum of **1** in DMSO (600 MHz), Figure S9: NOESY spectrum of **1** in DMSO (600 MHz).
