# Supplementary material for: Secondary Metabolites from the Culture of the Marine-derived Fungus Paradendryphiella salina PC 362H and Evaluation of the Anticancer Activity of Its Metabolite Hyalodendrin
Source: Mar Drugs. 2020 Apr 3;18(4):191. doi: 10.3390/md18040191 (PMC7230232; doi:10.3390/md18040191)
Supplement: Supplementary file 1 [file marinedrugs-18-00191-s001.pdf]

SUPPLEMENTARY INFORMATIONS

Supplementary materials and methods.

Fungal material

The fungus was isolated in January 2012 as an endophyte from the holdfast of *P. caniculata* harvested in Roscoff (Britany, France). Both morphological assessment and internal transcribed spacer (ITS) sequencing were performed to identify it as *Paradendryphiella salina.* It is now maintained at the MCAM culture collection (Muséum National d'Histoire Naturelle, Paris) under the number PC 362H.

Antiplasmodial assay

The anti-plasmodial activity was evaluated against the chloroquine-resistant FcB1/Colombia strain of *Plasmodium falciparum*. The assay was performed using the method of Desjardins (Desjardins et al., 1979). Extracts and pure compounds were diluted in Dimethyl Sulfoxyde (DMSO) and added to the culture medium (final concentration: 20 μg/mL) in 96-well microplates. Asynchronous parasite cultures were then added (1 % parasitemia and 1 % final hematocrite), and plates were maintained for 24 hours at 37 °C in a candle jar. [^{3}H]hypoxanthine (0.5 μCi) was subsequently added to each well, and parasites were maintained for an additional 24 hours. After a cycle of freezing and thawing, the cells were harvested from each well onto glass fiber filters, and the dried filters were counted in a scintillation counter. The growth inhibition for each well was determined by comparison of the radioactivity incorporated into the treated culture with that in the control culture maintained on the same plate. Chloroquine was used as positive control. Experiments were performed in triplicate.

Immunofluorescence

MCF7-Sh-WISP2 cells were incubated with the indicated concentrations of **3** at 37 °C. SN-38 (7-Ethyl-10-hydroxycamptothecin) was used as a positive control. Cells were fixed in PBS plus 4 % formaldehyde for 20 min and permeabilized for 15 minutes at room temperature with PBS supplemented with 0.5 % triton X100 (Sigma, Lyon, France). Incubation with primary and secondary antibodies was carried out for 1 hour at room temperature in PBS plus 1 % bovine serum albumin and 0.2 % gelatin fish (Sigma). The antigens were revealed by using the indicated primary antibodies. γ-H2AX antibody (# 05-636, Millipore, Lake Placid, NY) was used at a dilution of 1:100; PRAS40 antibody (# 701058, Life Technologies, Carlsbad, California) was used at a dilution of 1:250. Fluorescent dye conjugated secondary antibodies (Jackson ImmunoResearch, Bar Harbor, ME) were used at a dilution of 1:200. Coverslips were then mounted with 4',6-diamidino-2-phenylindole (DAPI) containing VECTASHIELD (# H-1200, Vector Laboratories) and sealed. Images were collected using a BX61 fluorescent microscope and cell F imaging software (Olympus).

Table S1. Evaluation of the 138 crude extracts on MCF7-Sh-WISP2 cell viability.

IC$_{50}$ (µg / mL)	MEA-ASW			Tubaki		
**Strain code**	**MCF7**	**MCF7-Sh-**	**3T3-F442A**	**MCF7**	**MCF7-Sh-**	**3T3-F442A**
LD8H	117,2	10,4	128,4	129,3	106,4	131,3
LD13H	215,7	89,2	160,0	42,3	28,0	> 250
LD14H	17,3	48,4	64,0	36,9	52,8	67,4
LD40H	> 250	> 250	> 250	94,7	86,4	103,5
LD41H	42,7	178,0	140,0	71,2	96,4	41,9
LD42H	51,5	90,8	175,7	103,5	72,8	144,0
AN44R	187,4	186,8	> 250	43,7	132,8	32,4
LD46H	190,4	195,2	131,3	68,8	108,0	126,4
LD50H	> 250	> 250	> 250	>250	> 250	> 250
LD53H	60,0	175,7	124,3	76,0	98,8	82,4
LD54H	213,0	213,6	173,3	> 250	170,4	136,8
LD69H	54,7	58,0	57,6	88,7	60,0	41,9
AN120R	8,0	8,5	3,7	1,7	3,7	0,9
AN122R	8,4	8,4	36,0	9,7	32,4	45,3
AN130T	> 250	176,8	166,4	68,8	180,0	133,7
AN131R	1,6	8,8	16,0	19,0	12,8	74,0
LD133T	174,7	> 250	> 250	94,0	140,0	156,0
LD144H	> 250	52,0	234,7	200,0	86,4	> 250
LD147H	32,5	48,4	122,9	52,0	38,0	68,7
LD149H	57,6	94,8	141,5	94,0	47,6	57,6
LD150F	0,3	2,7	6,3	2,4	3,4	36,8
LD155H	49,3	86,0	156,0	83,7	82,0	86,7
LD187R	> 250	> 250	183,0	187,4	> 250	70,7
LD294F	1,7	24,4	17,3	0,3	16,2	3,3
AN312R	4,4	21,0	32,5	4,5	5,4	16,0
AN325T	1,5	0,4	3,7	1,8	1,2	3,8
LD326H	16,6	75,2	126,4	57,6	52,8	41,9
LD327H	0,9	0,3	0,7	0,5	0,1	0,1
SL328T	80,7	94,0	130,2	110,4	182,4	101,5
AN329T	37,8	58,4	142,5	> 250	174,4	138,0
LD330H	40,4	32,8	50,7	71,2	56,8	21,0
LD331H	> 250	> 250	195,3	> 250	> 250	210,0
SL332T	4,2	3,0	3,3	61,0	111,2	50,7
SL333T	37,8	28,0	59,2	64,6	87,2	84,5
PC334T	58,8	84,0	152,7	59,6	123,6	77,6
PC337H	32,2	54,8	156,0	82,3	216,4	139,2
PC359H	1,6	0,9	1,6	32,8	42,8	62,5
PC361H	45,1	80,0	130,9	43,7	55,2	74,0
PC362H	0,4	0,2	0,5	8,3	10,0	16,7
LD366H	113,3	219,6	> 250	95,3	216,0	> 250
AN397T	24,6	75,2	44,3	41,1	148,8	139,2
PC398R	81,3	72,4	131,8	38,9	101,6	56,8
SL405T	44,5	86,8	170,7	93,7	140,4	132,3
SL408T	162,5	148,0	> 250	41,6	128,8	79,2
SL411T	32,3	86,8	92,3	34,0	45,6	43,2
SL428T	24,6	84,8	53,3	36,5	160,0	60,0
LD447H	67,4	65,2	90,7	38,9	131,2	4,5
LD448H	243,5	> 250	215,0	200,0	> 250	> 250
LD449H	9,6	18,0	56,0	65,2	94,4	74,0
LD450H	240,0	> 250	> 250	> 250	200,0	98,0
SL457T	> 250	204,4	165,1	89,1	128,8	50,4
SL464T	> 250	> 250	> 250	> 250	114,0	> 250
SL467T	82,4	153,2	72,7	108,0	235,2	161,3
SL468T	56,4	> 250	114,7	129,1	172,8	43,5
SL469T	88,2	22,8	37,6	2,3	2,8	4,7
SL470T	56,8	175,0	139,6	57,2	85,6	65,7
SL472T	> 250	> 250	> 250	226,8	245,2	142,0
SL473T	80,8	104,4	124,0	70,4	66,8	62,0
SL474T	> 250	168,8	200,0	88,7	122,8	54,3
AN480T	40,8	120,0	112,7	128,7	144,0	56,4
LD481H	0,3	0,4	0,3	0,3	0,9	0,2
LD482H	142,5	91,2	> 250	125,6	130,8	> 250
LD534H	16,6	22,3	63,5	116,0	90,0	160,0
LD535H	232,7	> 250	172,0	152,7	243,6	88,7
PC538R	138,8	222,8	131,1	89,3	178,4	104,0
SL539T	> 250	> 250	200,0	209,8	> 250	72,0
SL540T	84,0	222,0	70,4	128,7	175,0	92,7
AN596H	214,3	50,4	142,0	68,8	39,2	82,1
PC611R	89,3	182,0	243,7	92,0	143,6	63,0

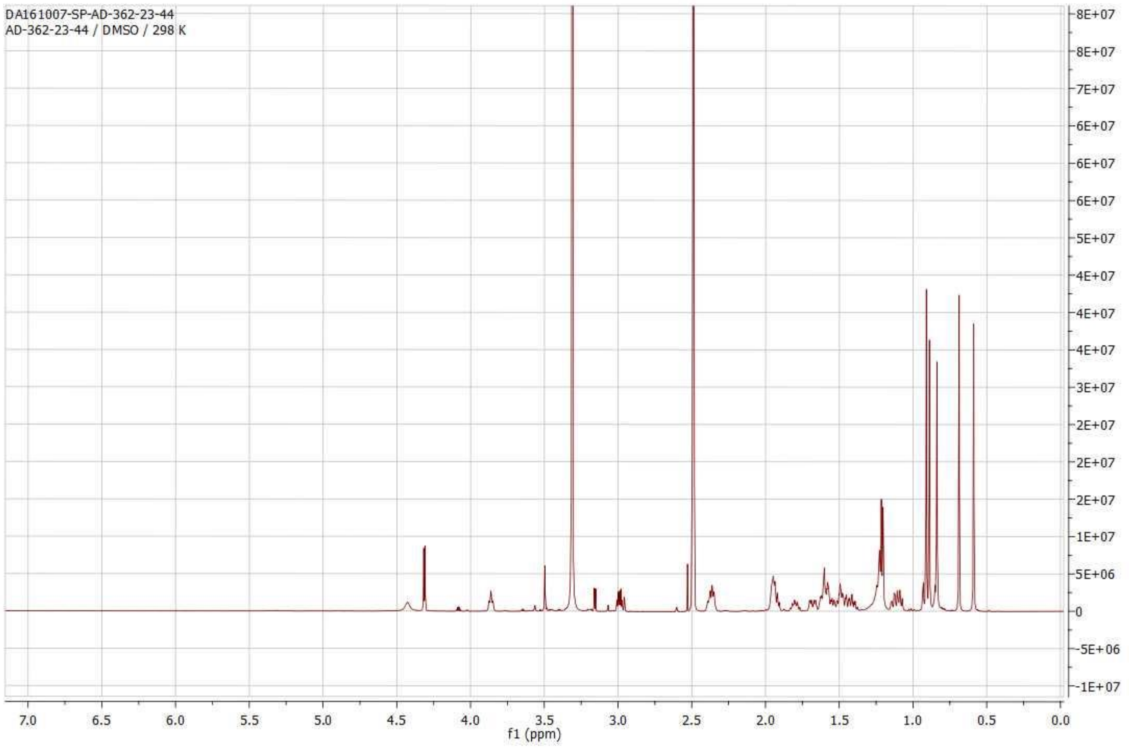

Figure S1. ^{1}H spectrum of **1** in DMSO (600 MHz).

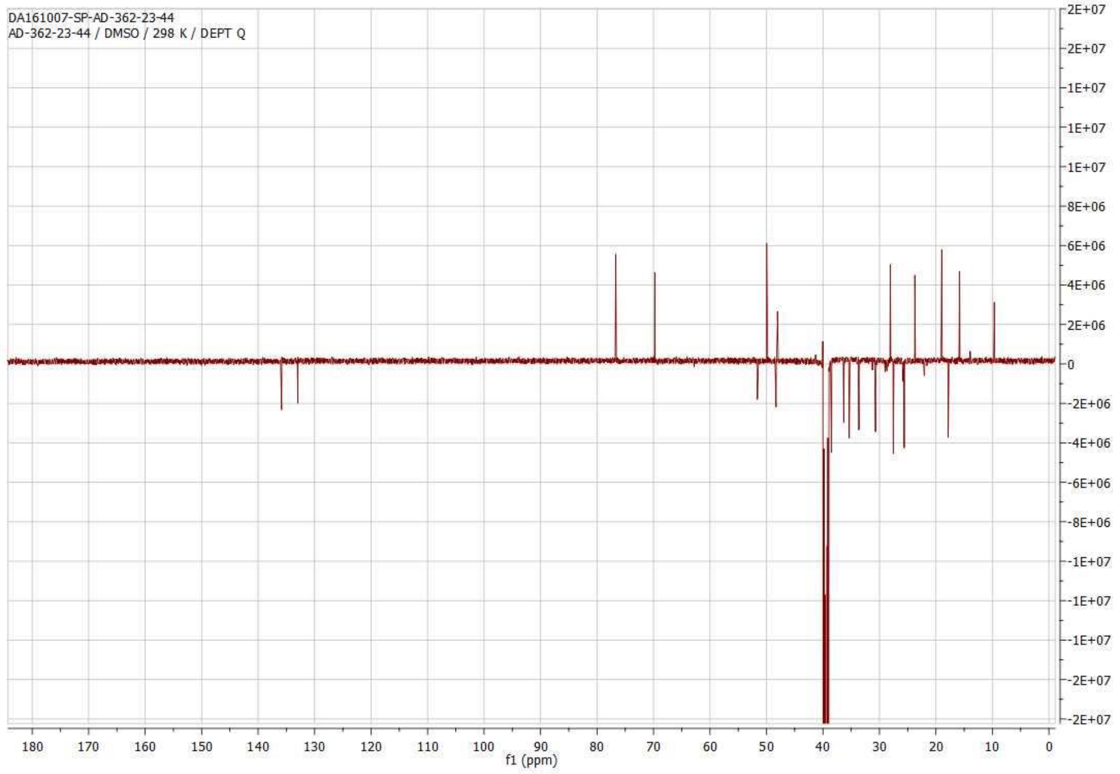

Figure S2. DEPT 135 spectrum of **1** in DMSO (150 MHz).

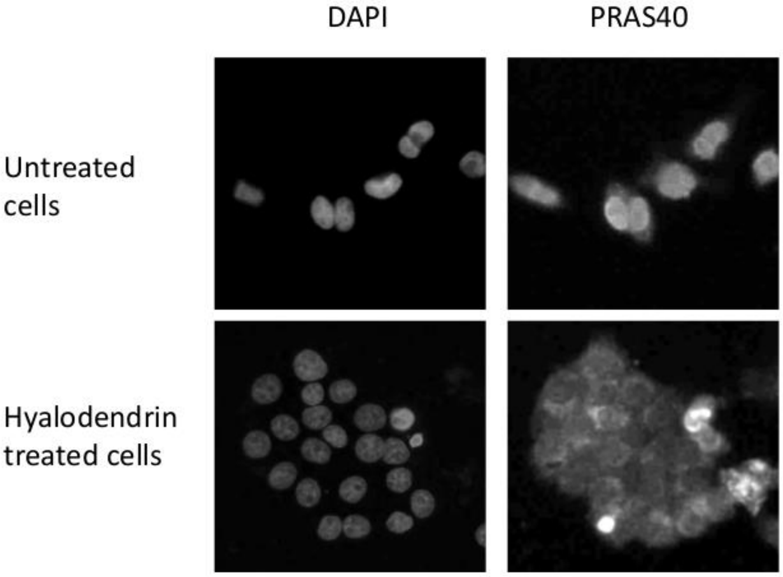

Figure S3. Subcellular localization of PRAS40 in MCF7-Sh-WISP2 cells exposed or not to compound **3**. Cells were treated or not with 1 μM of **3** (hyalodendrin) for 72 hours and immunolabelled with a PRAS40 targeted antibody. DAPI labelling of DNA are shown on the left panels; PRAS40 immunolabelling are shown on the right ones.

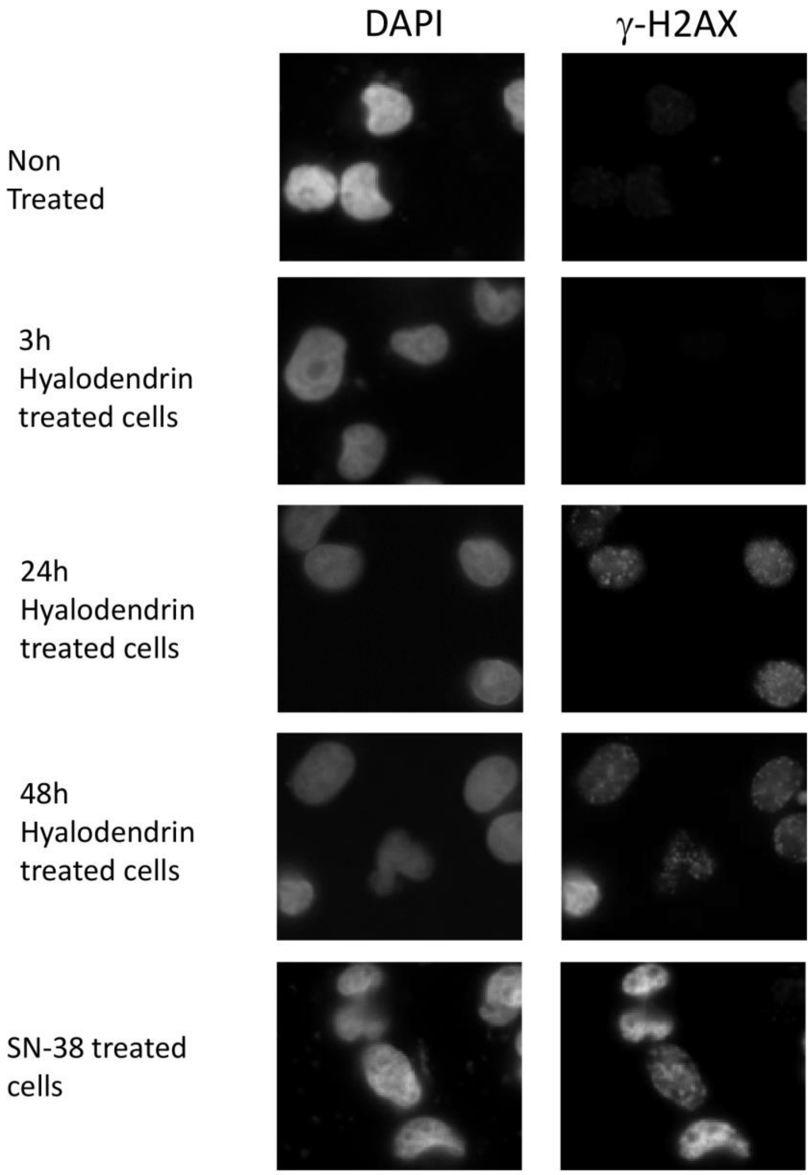

Figure S4. γ-H2AX foci formation in MCF7-Sh-WISP2 cells exposed or not to compound **3**. Cells were treated or not with 1 μM of **3** (hyalodendrin) for 0, 3, 24 and 48 hours and immunolabelled with a γ-H2AX targeted antibody. SN-38-treated MCF7-Sh-WISP2 cells were used as a positive control (1 hour exposure to 200 nM of SN-38). DAPI labelling of DNA are shown on the left panels; γ-H2AX immunolabelling are shown on the right panels.

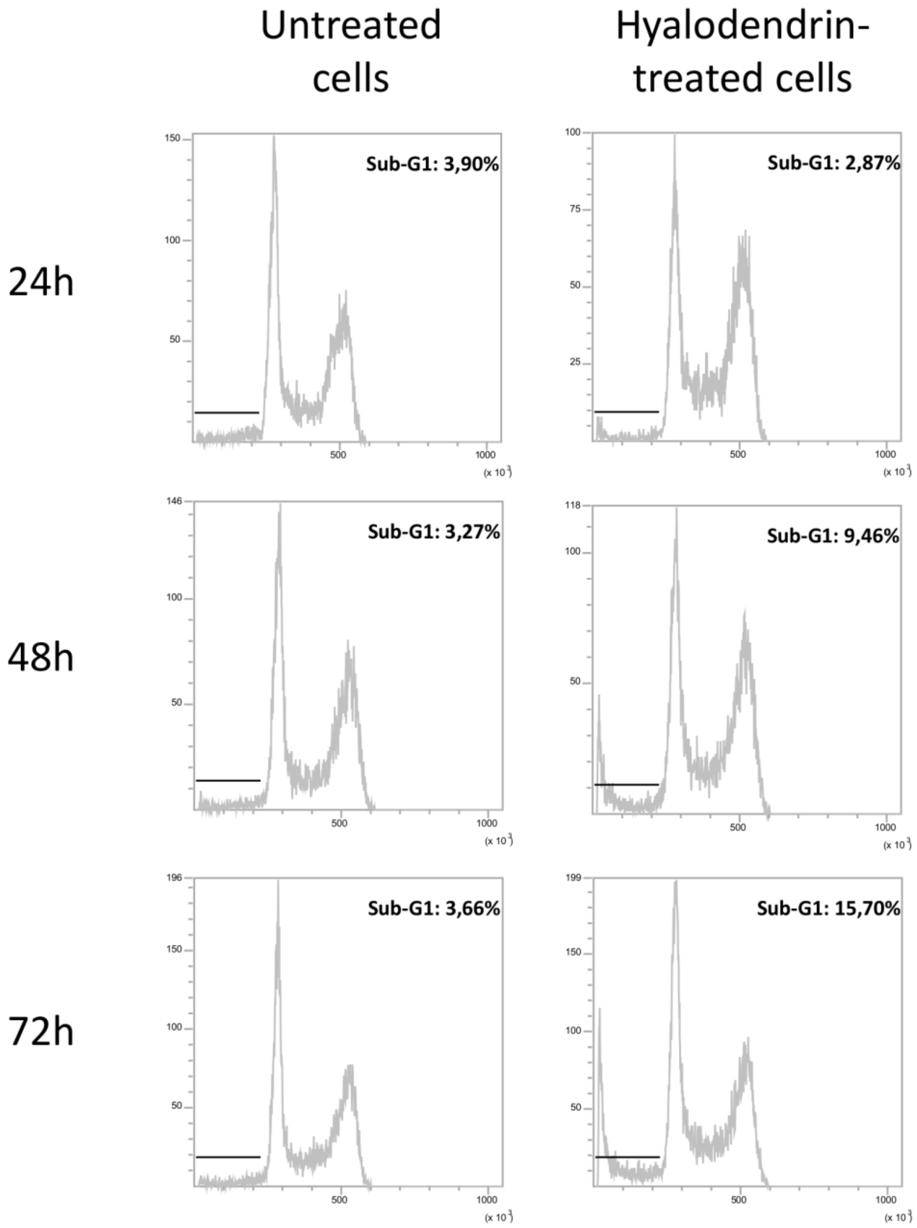

Figure S5. Cell cycle progression of MCF7-Sh-WISP2 cells in response to compound **3** exposure. After treatment with 380 nM of **3** (hyalodendrin) (corresponding to 1.9 10^{-5} nmol of drug per cell), cells were collected, counted and fixed with 70 % ethanol. Cells were then stained with 40 μg/mL propidium iodide (PI) (Biolegend) containing RNaseA (100 μg/mL), prior analysis on a Gallios flow cytometer (Beckman Coulter, Inc, Villepinte, France). The results were analyzed using the Venturi One V6 software (Applied Cytometry).

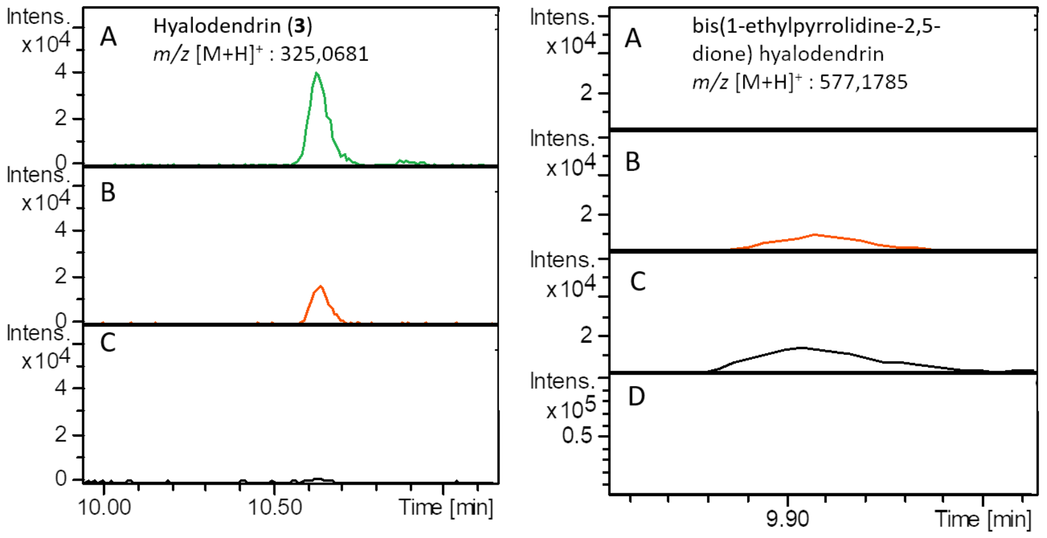

Figure S6. Extracted ion chromatograms of **3** (hyalodendrin) at *m/z* $[M+H]^+$ 325,0681 and N-ethylmaleimide conjugated compound **3** (N-ethylmaleimide hyalodendrin) at *m/z* $[M+H]^+$ 577,1785 resulting from *in vitro* alkylation of **3** in the presence of Trx system after addition of N-ethylmaleimide at two different incubation times. (A) **3** (hyalodendrin) without Trx system, (B) **3** (hyalodendrin) in the presence of Trx system and quenched with N-ethylmaleimide at 6 minutes, (C) **3** (hyalodendrin) in the presence of Trx system and quenched with N-ethylmaleimide at 25 minutes, (D) Trx system alone.

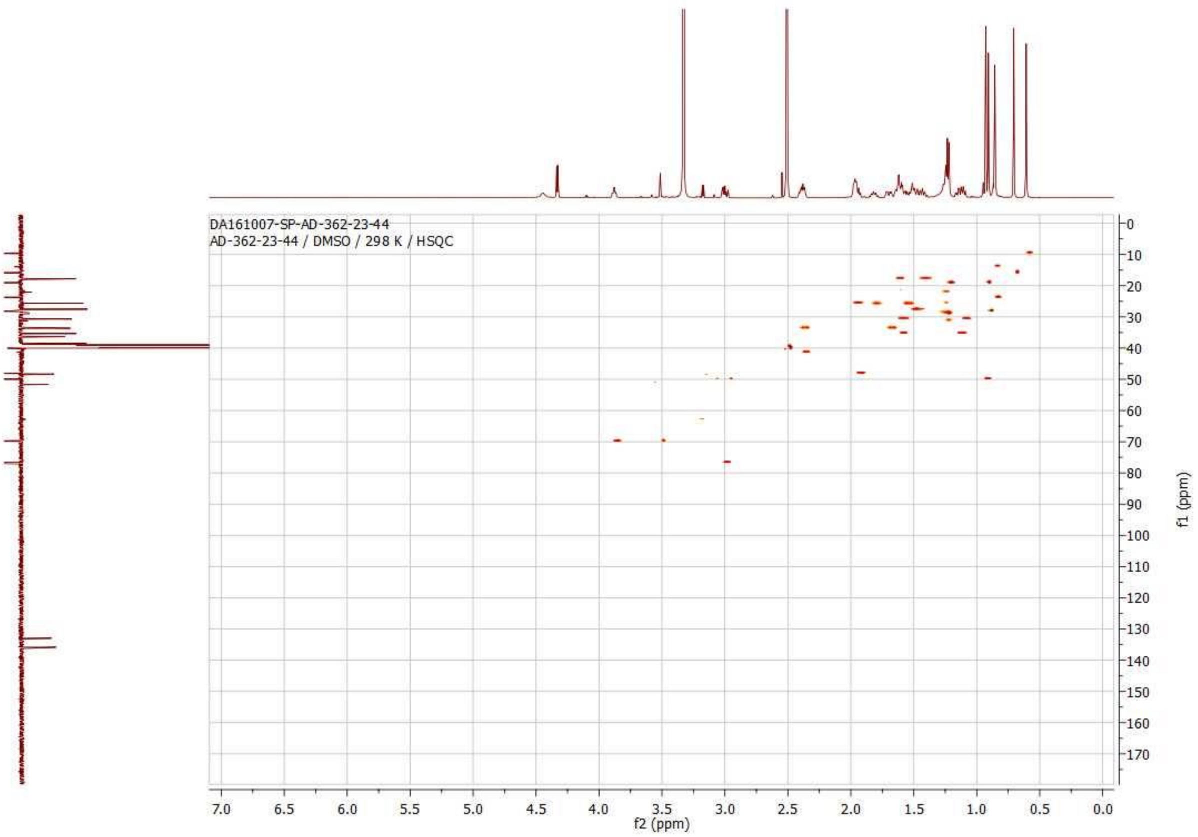

Figure S7. HSQC spectrum of **1** in DMSO (600 MHz).

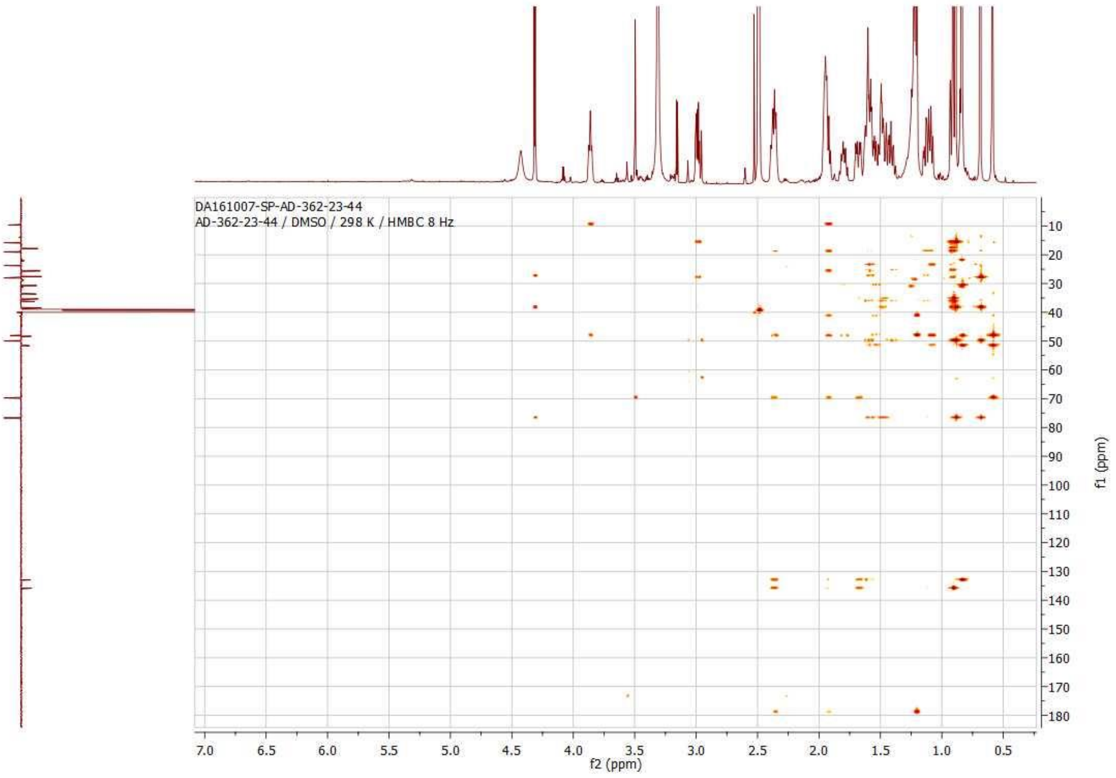

Figure S8. HMBC spectrum of **1** in DMSO (600 MHz).

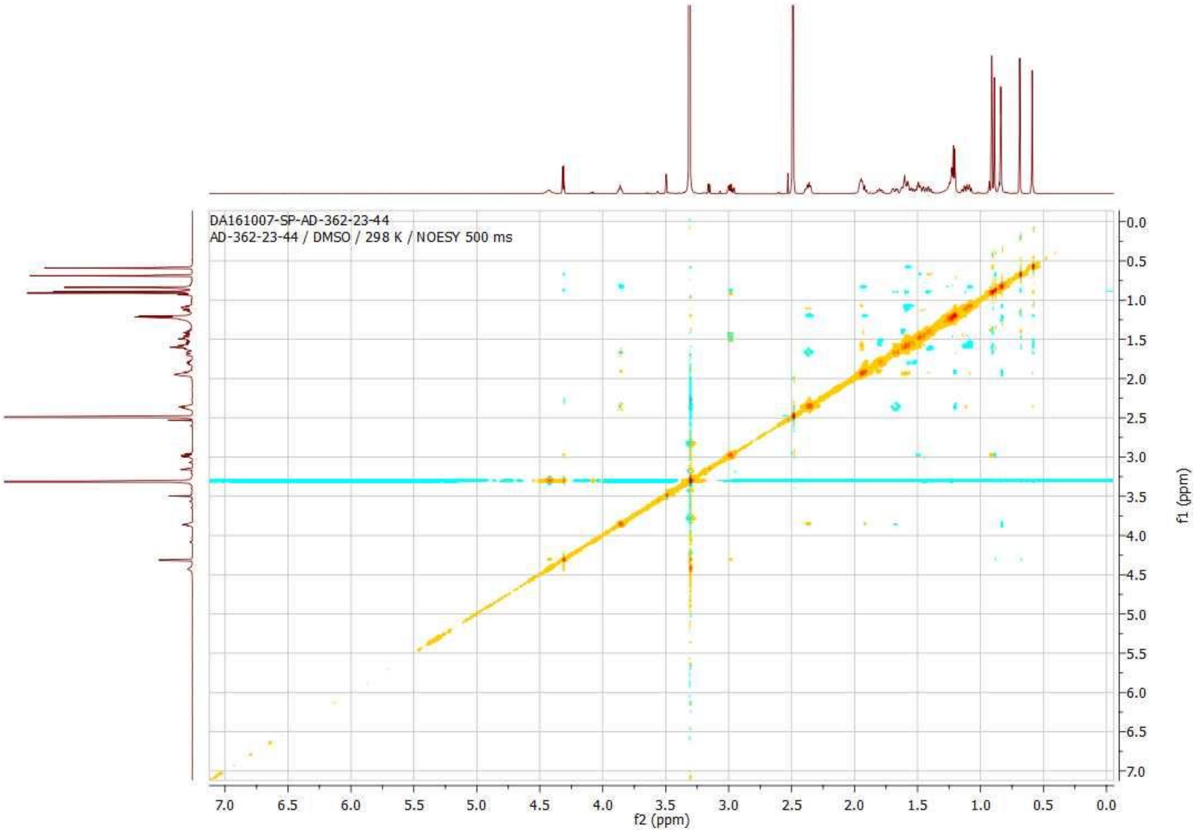

Figure S9. NOESY spectrum of **1** in DMSO (600 MHz).

Table S2. ^{1}H and ^{13}C NMR data of **2** in CD_3OD (^{1}H 600 MHz, ^{13}C 150 MHz).

**Pos.**	**δC, type**	**δH *mult.* (*J* in Hz)**	**Pos.**	**δC, type**	**δH *mult.* (*J* in Hz)**
**1**	36.5, CH_2	0.99, td (14.2, 3.4); 1.80, m	**15**	24.23, CH_2	1.11, m; 1.75, m
**2**	40.5, CH_2	1.64, m; 2.28 m	**16**	28.7, CH_2	1.31, m, 1.90, m
**3**	72.9, CH	3.37, m	**17**	48.1, CH	1.88, m
**4**	29.6, CH_2	1.58, m	**CH_3-18**	13.0, CH_3	0.72, s
**5**	43.2, CH	1.38, m	**CH_3-19**	23.2, CH_3	0.92, s
**6**	35.9, CH_2	1.53, m; 1.96, m	**20**	36.8, CH	1.44, m
**7**	69.1, CH	3.80, br q (2.8)	**CH_3-21**	17.7, CH_3	1.04, d (6.5)
**8**	41.0, CH	1.56 (m)	**22**	33.1, CH_2	1.36, m; 1.81, m
**9**	27.9, CH	2.25 (m)	**23**	33.7, CH_2	2.19, m; 2.32, m,
**10**	35.9, C	-	**24**	177.4, C	-
**11**	31.2, CH_2	1.42, m; 1.60, m	**25**	41.8, CH_2	3.92, s
**12**	74.1, CH	3.96, t (2.6)	**26**	171.9	-
**13**	47.5, C	-	**CH_3-27**	52.5	3.72, s
**14**	43.0, CH	2.00, m			

Table S3. NMR spectra (^{1}H and ^{13}C) for compound **3** and **4** in $CDCl_3$

Compound (3)
^{1}H-NMR ($CDCl_3$, 600 MHz):
7.34-7.28 (5H, m, H-10, H-11, H-12, H-13, H-14), 4.41 (1H, d, J = 12.5 Hz, H-7a), 4.33 (1H, d, J = 12.5, H-7b), 4.11/3.65 (1H, d, J = 15.8 and 1H, d, J = 15.8 , CH_2-Ph), 3.23 and 3.00 (3H, s, 1-NCH_3 and 3H, s, 4-NCH_3).

^{13}C-NMR ($CDCl_3$, 600 MHz):
166.9 (C, C-5), 165.6 (C, C-2), 134.02 (C, C-9), 129.2 (2CH, C-10, C-14), 129.01 (2CH, C-11, C-13), 127.3 (CH,C-12), 75.6 (C, C-3), 75.3 (C, C-6), 61.1 (CH_2, C-7), 36.7 (CH_2, C-8), 28.7 and 27.6 (CH_3, 4-NCH_3 and CH_3, 4-NCH_3).

Compound (4)
^{1}H-NMR ($CDCl_3$, 600 MHz):
7.22 (3H, m, H-11, H-12, H-13), 7.08 (2H, m, H-10, H-14), 3.82 (1H, d, J = 11.9 Hz, H-7a), 3.71 (1H, d, J = 13.9, CH_2-Ph), 3.16 (1H, d, J = 11.9, H-7b), 3.11 (1H, d, J = 13.9, CH_2-Ph), 3.23 and 3.00 (3H, s, 1-NCH_3 and 3H, s, 4-NCH_3), 2.27 and 2.10 (1H, s, 3H, s, 6-SCH_3 and 3H, s, 6-SCH_3)

^{13}C-NMR ($CDCl_3$, 600 MHz): δ= 165.3 (C, C-5), 165.1 (C, C-2), 133.8 (C, C-9), 129.8 (2CH, C-10, C-14), 128.5 (2CH, C-11, C-13), 127.5 (CH,C-12), 73.4 (C, C-3), 71.4 (C, C-6), 63.72 (CH_2, C-7), 42.1 (CH_2, C-8), 30.8 and 29.2 (CH_3, 4-NCH_3 and CH_3, 4-NCH_3), 14.1 and 13.2 (CH_3,1-NCH_3 and CH_3,6-SCH_3)
